# Most valued author and reviewer in 2021

**DOI:** 10.1080/13814788.2022.2079850

**Published:** 2022-06-06

**Authors:** Henk van Weert, Jelle Stoffers

The *European Journal of General Practice* (EJGP) is the scientific journal of WONCA Europe. WONCA is an acronym comprising the first five initials of the World Organisation of National Colleges, Academies and Academic Associations of General Practitioners/Family Physicians. WONCA not only represents the discipline of general practice/family medicine and its professionals – the general practitioners/family physicians – but also academic General Practice. This dual representation translates into a firm mission statement: ‘to improve the quality of life of people through fostering high standards of care in general practice/family medicine’ [1].

One of the means to reach this goal is the EJGP. The Journal tries to support the mission by publishing high-quality research – conducted by general practitioners or other researchers in general practice – which could help to improve the quality of life of the people we are serving, i.e. our patients. This goal forms the basis of our editorial policy to publish research that matters to our patients directly or indirectly. We believe that the health and quality of life of our patients is best served by GPs who provide high quality care. To this end, GPs must be not only well trained, organised and equipped but also be critical.

## Volume 27, 2021

Every year, the editors of the EJGP evaluate the past volume. In 2021, we published 32 original articles – eight of which were conducted with qualitative research methods –, five (systematic) review articles and four Editorials. Apparently, our Series on Qualitative Research Methodology published from 2017 onwards (our most successful project in terms of page views and downloads) still reverberates. It is good that GPs are doing qualitative research and we are pleased that we can support this with our Series [2]. Qualitative research is the GP's weapon in studying something new, something that raises questions or appears as unusual. A few years ago, when no one had thought of Q fever, qualitative research shed the first light on the mystery of an increasing number of patients with a strange form of pneumonia in the south of the Netherlands [3].

We expected a wave of manuscripts on COVID-19 since we had published two Editorials on the subject. That expectation came true. We published six articles on COVID-19 but had to reject many more manuscripts. The Editorial by Athina Tatsioni et al. evaluates the reasons for these rejections [4].

## Most valued paper

In 2021 too, authors came from many countries within and outside Europe. Like every year, each editor selected five articles that he or she found to be the most valuable (and whose scientific quality was rated at least as 'good'). We looked at relevance for general practice (family medicine, primary care), originality, methodology and possible impact on practice or research. That led to a paper on COVID-19 by Anne Holm of Copenhagen as the editors-reviewed most valuable paper of 2021 [5]. Anne Holm is a GP trying to solve a problem she encountered in the first year of the pandemic: what would be the best treatment for out-of-hospital COVID patients? A very relevant question at the time, for which she used the appropriate methodology. She and her colleagues did a scoping review and (unexpectedly) found that answers were hard to find because published research focussed (again!) on the hospitals. As GPs, we knew about the struggles in society and general practice in the fight against COVID-19 but apparently could not turn our experience into science. A hard lesson for the future.

You can read these and other published articles in the EJGP [6].

## Best valued reviewer

Finally, we would like to express our thanks to all reviewers who advised us during 2021. We really appreciate their help! Because reviewers are just as important to us as authors, the editors also chose a reviewer who deserves to be put in the spotlight. We want to mention explicitly that the editor-in-chief has not played an active role in the selection process this year, as the best-valued reviewer of 2021 works in his department in Maastricht: Dr Piet Portegijs. Piet Portegijs is a trained general practitioner who specialised in epidemiology. He (more often) provides us with to-the-point reviews with constructive explanations and clear suggestions for the authors and editors. Thanks to people like Piet, we can do our job well.

## Create and update your EJGP account

Although our database of authors and reviewers steadily increases, the editors find it challenging to select referees within the Journal’s deadlines. Therefore, we sincerely hope that all reviewers want to continue their review work. Please, update your EJGP account, e.g. specify your expertise, and visit https://mc.manuscriptcentral.com/ejgp to modify your profile. In addition, we invite all readers of the EJGP interested in reviewing manuscripts for this Journal to visit https://mc.manuscriptcentral.com/ejgp and register as a ‘new user’, i.e. to complete the details of their expertise.
